# In-situ tool wear condition monitoring during the end milling process based on dynamic mode and abnormal evaluation

**DOI:** 10.1038/s41598-024-63865-4

**Published:** 2024-06-05

**Authors:** Min Chen, Jianwei Mao, Yu Fu, Xin Liu, Yuqing Zhou, Weifang Sun

**Affiliations:** 1 Zhejiang Dewei Cemented Carbide Manufacturing Co., Ltd., Wenzhou, 325699 China; 2 College of Mechanical and Electrical Engineering, Wenzhou University, Wenzhou, 325035 China; 3 College of Mechanical and Electrical Engineering, Jiaxing Nanhu University, Jiaxing, 314001 China

**Keywords:** Dynamic mode decomposition, Tool wear, Condition monitoring, Abnormal evaluation, Graph similarity, Information theory and computation, Electrical and electronic engineering, Mechanical engineering

## Abstract

Rapid tool wear conditions during the manufacturing process are crucial for the enhancement of product quality. As an extension of our recent works, in this research, a generic in-situ tool wear condition monitoring during the end milling process based on dynamic mode and abnormal evaluation is proposed. With the engagement of dynamic mode decomposition, the real-time response of the sensing physical quantity during the end milling process can be predicted. Besides, by constructing the graph structure of the time series and calculating the difference between the predicted signal and the real-time signal, the anomaly can be acquired. Meanwhile, the tool wear state during the end milling process can be successfully evaluated. The proposed method is validated in milling tool wear experiments and received positive results (the mean relative error is recorded as 0.0507). The research, therefore, paves a new way to realize the in-situ tool wear condition monitoring.

## Introduction

Condition monitoring and fault diagnosis for computer numerical control (CNC) machines have been widely investigated in recent years and achieved great progress^1,2^. As a crucial component used to remove materials from the workpiece, the cutting tool’s running state will inevitably influence the surface quality of the final part, as well as the cutting process stability^3,4^. Therefore, rapid tool operating state estimation is important to maintaining the machining performance of the cutting system, preventing workpiece scrap and operator injury.

**Figure 1:**
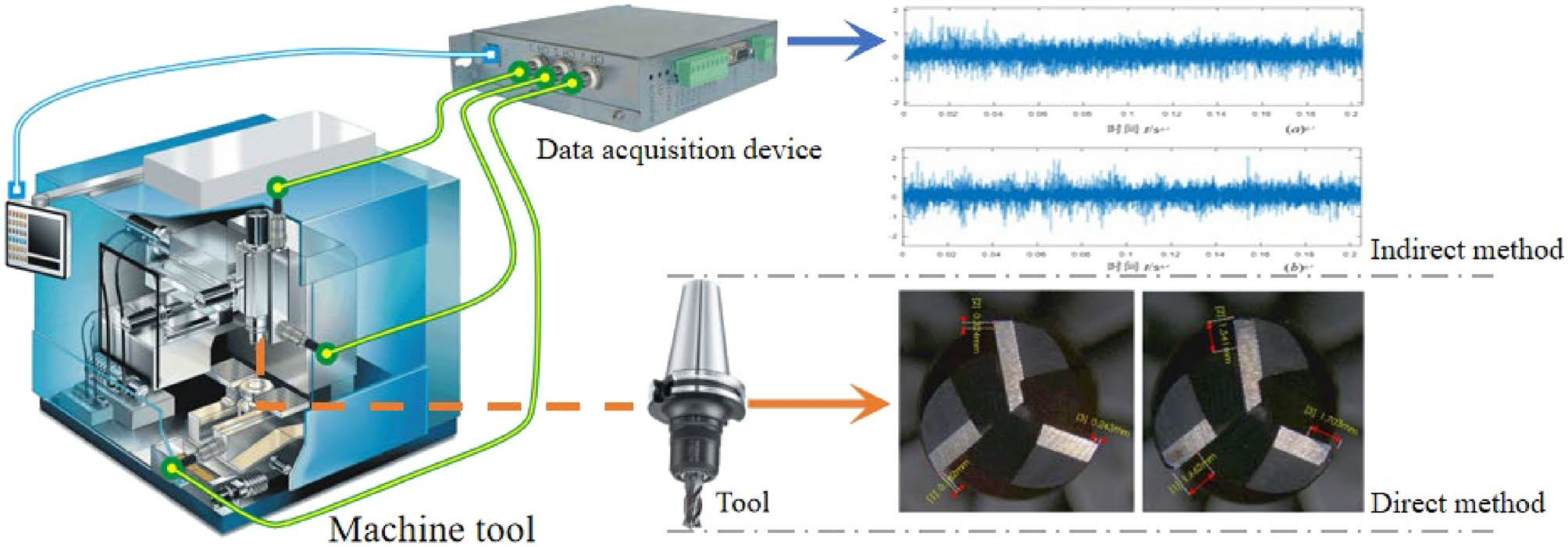
Tool condition monitoring methods.

To realize rapid tool operating state estimation, considerable research efforts have been devoted. In such works, based on the physical location and measurement object of the sensor, those methods can be divided into direct methods and indirect methods^5^. As can be seen in Fig. 1, direct methods can directly acquire the digital image of cutting edges and evaluate the tool wear state accordingly. For the indirect methods, dynamic signals during the manufacturing process can be sampled across the sensor mounted on the workpiece, spindles, or other components^6,7^. The tool wear state can be estimated indirectly based on the acquired signals.

Benefiting from the implementation convenience, direct methods were successfully demonstrated in a number of studies, and the robustness of the methods is also testified. To realize the tool condition monitoring, considerable attention has been paid to evolution mechanism exploration and attempted to identify the service state based on their characteristic information. Among them, feature extraction based on sparse measure optimization has emerged as an interesting candidate for identifying the health state of mechanical systems. Based on the specific requirement, via the feature extraction methods, the mathematical model and the response characteristics can be investigated. After that, the optimal filter bank is obtained through iterative or non-iterative methods to achieve explicit representation of features. To address the problem that traditional tool wear prediction methods rely on the experience and knowledge of experts, Yang et al.^8^ proposed a new tool wear prediction method based on local features and global dependencies. Focus on the weak fault detection of the rolling bearing in strong noise conditions, Deng et al.^9^ propose a novel fault diagnosis method with an improved empirical wavelet transform (EWT) and the maximum correlated kurtosis deconvolution (MCKD). To address the low efficiency of iterative solutions during the MCKD process, Mcdonald et al.^10^ proposed a non-iterative deconvolution method to directly acquire the optimal filter coefficient and successfully apply it in related scenarios. These researches provide the theoretical basis for system state identification. However, with the increasing complexity and systematization of mechanical equipment, the failure modes also become complex and variable, which leads to the instability of the proposed methods. In addition, traditional sparse measure optimization methods strongly rely on the prior knowledge of professional technicians and diagnostic experts in the diagnostic process (such as system structure, fault frequency, etc.)^11^, which restricts the applicability of these excellent methods in a wider range of engineering application scenarios.

With the rapid development of machine learning technology, artificial intelligence (AI) based fault diagnosis and prediction have increasingly become an important strategy for equipment safety and service monitoring^12^. Via related intelligent algorithms, the data-driven diagnostic method can adaptively identify equipment operation status information from existing data without the need of prior knowledge for professional technicians^13^. With an edge-labeling graph neural network method, Zhi et al.^14^ propose a tool for wear condition monitoring using wear images which suitable for small sample conditions. Mishra et al.^15^ developed a tool condition estimation method during the precision machining process with the unsupervised approach. However, data-driven methods are inevitably influenced by the distribution of training data, which may lead to data bias in the training model. Combining the sparrow search algorithm, Li et al.^16^ developed a CNN-BiLSTM-based neural network to effectively predict sea level heights. Therefore, if the equipment status characteristics can be effectively re-characterized through a simple method, it is expected to overcome related shortcomings and achieve robust identification of its service performance.

Recently, time-domain-based CM-FD methods have been intensively investigated and successfully applied in some application scenarios^17^. Among them, the graph-based method enjoys the merits of anomalies quantitative evaluation and approximate shift-invariance^18,19^. However, it remains challenging to establish the adjacency matrix in a short time which might threaten the online evaluation reliability^20^. To overcome the potential drawbacks, as an extension of our works, Shiliang Feng et al.^21^ proposed a time-domain signal-driven mechanical system state description method and validated in some typical mechanical experiments.

During the manufacturing process, rapid tool wear might unpredictably occur, especially for hard-to-machining materials (e.g. nickel-based alloy or titanium alloy). The rapid tool wear will greatly affect the durability of cutting tools and the integrity of machining surfaces. To effectively trace the tool wear dynamic variation and avoid rapid deterioration of surface integrity, it is crucial to predict the short-term time-series response and estimate the tool wear status in advance. However, very little optimization work has been carried out on the dynamic evaluation of the tool wear state based on the predicted short-term time series.

Focus on the drawbacks and the existing research gap mentioned above, inspired by the time-domain-based CM-FD methods, in this research, a tool service state evaluation method that does not rely on any prior knowledge is proposed. With dynamic mode decomposition, the time series in the next snapshot can be predicted. Furthermore, based on graph structure, the anomalies of the tool wear state can be identified. The proposed strategy enjoys the merit of short-time time series prediction and offers exciting opportunities for rapid monitoring of tool wear state. The main structure of the manuscript is summarized as follows. A brief description of the proposed data prediction based on the dynamic mode decomposition method is listed in “Data prediction based on dynamic mode decomposition method” section. “Anomalies identification for time-series” section presents the proposed anomaly identification for time series. The In-situ tool wear condition monitoring is summarized in “The proposed in-situ tool wear condition monitoring method” section. The validation experiment of the proposed tool wear estimation method is listed in “Experimental investigation” section. The conclusion of the manuscript is listed in “Conclusion” section.

## Data prediction based on dynamic mode decomposition method

As a typical fluid dynamics analysis method, benefiting from the extraordinary spatiotemporal feature presentation ability (decomposing complex flow processes into low-rank spatiotemporal features), dynamic mode decomposition has lately received great attention. Because the decomposition does not rely on any given dynamic model, the method is suitable for dynamic process description. In this section, dynamic mode decomposition is employed for short-term time-series prediction.

### Model establishment

After equally resampling from the temporal signals, a multivariate time series can be acquired. Assuming the combined multivariate time series is composed of *M* temporal signals with a length of *T*, the expression at time *t* can be expressed as^22^:

1 $$ x_{t} = Ax_{t - 1} + \varepsilon_{t} , $$

where *A* is the Koopman matrix (coefficient matrix in vector autoregression process) with a dimension of *M* × *M*, and *ε* is the residual term.

Similar, by arranging the *T* snapshots into two large data matrices:

2 $$ \left\{ {\begin{array}{*{20}c} {X_{1} = \left[ {\begin{array}{*{20}c} | & | & {} & | \\ {x_{1} } & {x_{2} } & \cdots & {x_{T - 1} } \\ | & | & {} & | \\ \end{array} } \right]} \\ {X_{2} = \left[ {\begin{array}{*{20}c} | & | & {} & | \\ {x_{2} } & {x_{3} } & \cdots & {x_{T\;\;\;} } \\ | & | & {} & | \\ \end{array} } \right]} \\ \end{array} } \right.. $$

The expression of the dynamic mode decomposition can be represented as:

3 $$ X_{2} \approx AX_{1} . $$

Equation (3) can be regarded as a vector autoregressive problem. If *A* can be regarded as the Koopman matrix in dynamic mode decomposition, a low-rank structure can be used for the approximation. For autoregressive problems, if it is necessary to calculate the coefficient matrix *A*, by minimizing the squared residual, the matrix can be acquired.

4 $$ \min_{A} \left\| {X_{2} - AX_{1} } \right\|_{F}^{2} . $$

### Model solution

To decrease the model calculation complexity, intrinsic orthogonal decomposition methods (e.g. singular value decomposition) are widely employed to map the high-dimensional variables to low dimension.

With singular value decomposition, the matrix ***X***_**1**_ can be decomposed by^23^:

5 $$ X_{1} = U\sum {V^{T} } , $$

where *U* is a *m*-order unitary matrix, *V* is a *n*-order unitary matrix, Σ is a non-negative real diagonal matrix with dimension of *m* × *n*. Generally, each eigenvector in *V* is called the right singular vector of *M*, each eigenvector in *U* is called the left singular vector, and the elements on the diagonal of *D* are called the singular values of *M*. When the singular values are arranged in descending order, a unique *D* can be determined.

If the matrix *X*_1_ is truncated for singular value decomposition with a rank of *r*, the Koopman matrix *A* can be approximated using the following matrix:

6 $$ \tilde{A} = U_{r}^{T} X_{2} V_{r} \Sigma_{r}^{ - 1} , $$

where matrix *U*_*r*_ ∈ ℝ^*M*×*r*^, *V*_*r*_ ∈ ℝ^(*T*−1)×*r*^, Σ_*r*_ ∈ ℝ^r×*r*^ are the truncation matrixes of the unitary matrix ***U***, the unitary matrix ***V***, the non-negative real diagonal matrix, respectively.

### Data prediction

If it is necessary to solve the modal of a matrix and analyze its spatiotemporal characteristics using it, the matrix can be decomposed into eigenvalues^24^:

7 $$ \tilde{A} = Q\Phi Q^{T} , $$

where $\Phi$ is a diagonal matrix (the diagonal elements are the corresponding eigenvalues), the matrix *Q* is composed by the eigenvectors. Therefore, eigenvalues and eigenvectors can be used to analyze and predict the complex spatiotemporal characteristics of the system.

The mode of the dynamic can be defined as:

8 $$ \psi = X_{2} V_{r} \Sigma_{r}^{ - 1} Q. $$

Therefore, the dynamic prediction of data can be represented as:

9 $$ X_{2} = \Psi \Phi \Psi^{\dag } X_{1} , $$

where the symbol † indicates the Moore Penrose generalized inverse operation.

## Anomalies identification for time-series

A graph mechanism based on temporal signals is proposed to identify anomalies in temporal data.

### Graph structure description

In recent years, a novel abnormal health status of equipment evaluation method using time-domain signals has been proposed and aroused wide concerns in mechanical systems^25,26^. Based on the graph structure in computer science, the internal feature structure of signals can be evaluated, and the health state of the equipment can also be evaluated accordingly.

According to the graph theoretical, a graph structure can be expressed by *G* = {*N*, *E*}, where *N* is the set of nodes and *E* is the set of connections. Among them, the node set can be used to describe different sampling points, and the connection set *E* is used to describe the connection strength between different nodes. The connection strength between different nodes is reversible, so the set of connections is clearly a symmetric matrix.

**Figure 2:**
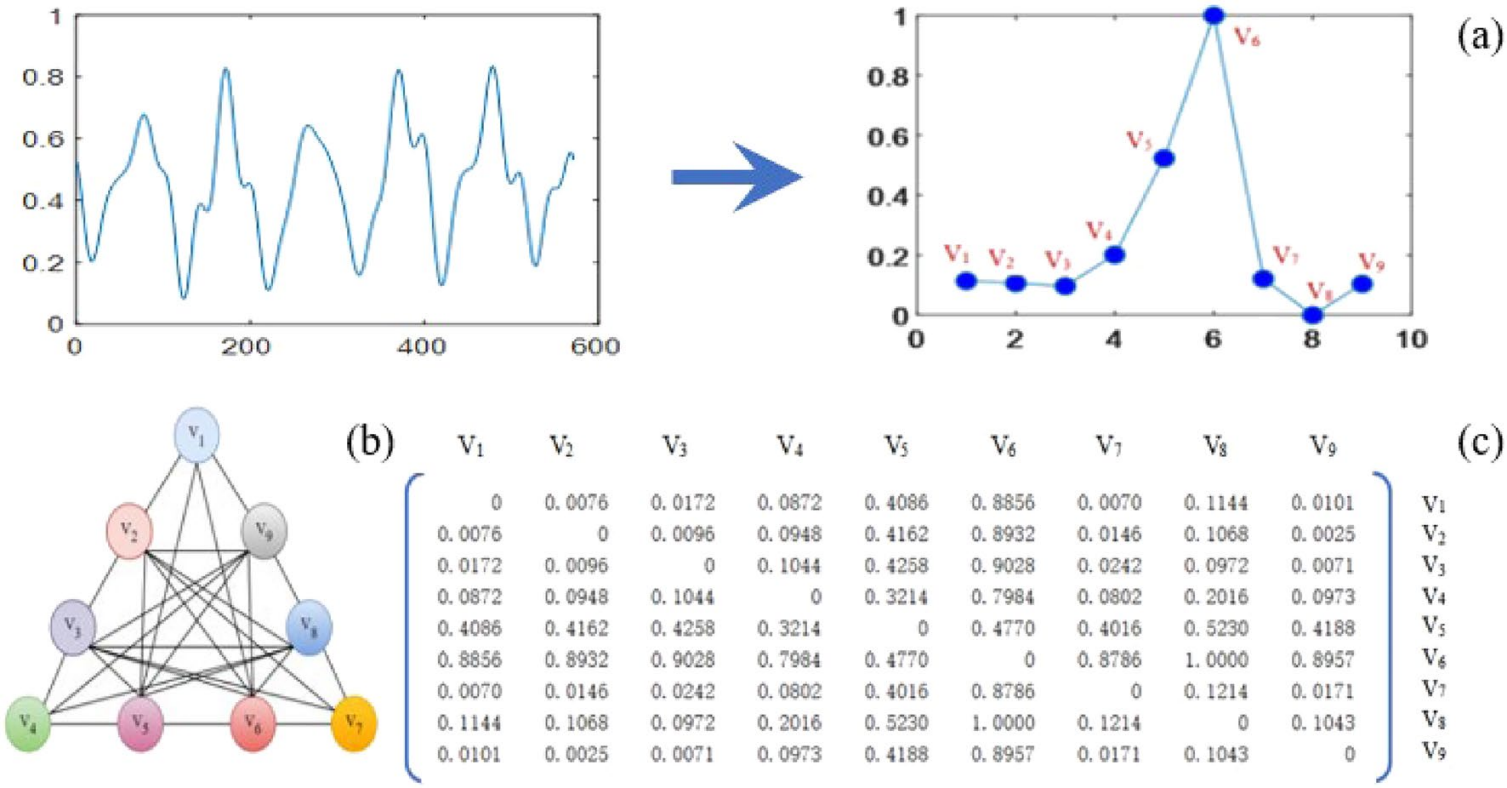
Adjacency matrix construction. (**a**) Node determination (**b**) Graph illustration (**c**) Adjacency matrix.

For any temporal signal (as shown in Fig. 2a), the adjacency matrix of its data segments can be expressed as a symmetric matrix (as shown in Fig. 2c). In this study, the connection strength between different nodes is described by the Euclidean distance between nodes (as shown in Fig. 2b). Therefore, the collected temporal signals can be reprojected into a set of adjacency matrices (detail of the process can be seen in Ref.^25^):

10 $$ F = \, \left\{ {G_{1} ,G_{2} , \, \ldots G_{n} } \right\} \, = \, \left\{ {X_{1} ,X_{2} , \, \ldots X_{n} } \right\}, $$

where *Xn* is the *n*-th symmetric matrix that constitutes the set of adjacency matrices.

### Anomalies identification

Based on the definition of graph structure, related researchers found that if the equipment state (i.e. operating state) is changed, the internal structure or parameters of its corresponding adjacency matrix will also change. Therefore, by evaluating the differences between the corresponding adjacency matrices, the dynamic characteristic of the equipment operation status can be monitored. The methods for anomaly identification can be summarized as follows.

The “standard template” ***X*** (in normal state) can be established from the sampled time series based on the traditional graph structure. The standard template matrix can be decomposed by^27^:

11 $$ X = \Gamma \Lambda \Gamma^{\prime}, $$

where $\Lambda$ is the eigenvalue matrix (diagonal elements are eigenvalues), ${{\varvec{\Gamma}}}$ is the eigenvector matrix (each column is the eigenvector of matrix *X*).

For a given test signal ***y***, the corresponding adjacency matrix *Y* can be established accordingly. Similarity, the adjacency matrix *Y* can be decomposed as^28^:

12 $$ \Gamma Y^{t} \Gamma^{\prime} = \Gamma (diag[Y^{t} ])\Gamma^{\prime} + \Gamma (non - diag[Y^{t} ])\Gamma^{\prime}, $$

where the symbol *diag*[.] represents the diagonal elements of the adjacency matrix, and *non–diag*[.] represents the non-diagonal elements of the adjacency matrix.

By evaluating the non-diagonal components, the similarity between the two signals can be evaluated. For in-situ tool condition monitoring problems, the Frobeniu norm of the non-diagonal can be directly employed for the tool wear evaluation.

## The proposed in-situ tool wear condition monitoring method

**Figure 3:**
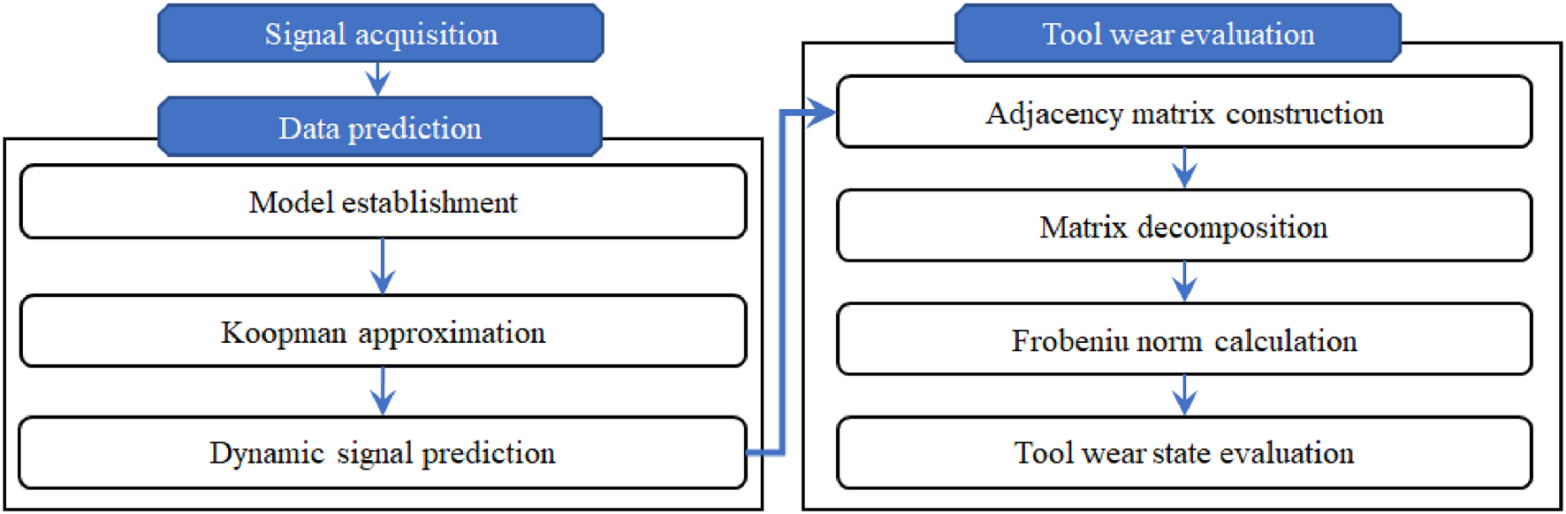
Flowchart of the proposed method.

Combining the dynamic mode decomposition and real-time prediction signal anomaly identification, a method for evaluating the wear status of machining tools without relying on any given prior knowledge is proposed in this research. The main process of this method can be described in Fig. 3, the specific step is listed as follows:

- *Step 1* Based on the sensor (near the cutting area) and the digital signal acquisition device, the dynamic signal which can reflect the tool service status information is recorded.
- *Step 2* By using the dynamic mode decomposition method established in “Data prediction based on dynamic mode decomposition method” section, the time-series signal in the next moment can be predicted.
- *Step 3* With the graph establishment approach (as shown in Eq. (10)), the graph structures of the current moment and the predicted signal can be constructed.
- *Step 4* Taking the acquired signal as the “standard template”, as shown in Eq. (12), the graph structure of the predicted signal can be decomposed.
- *Step 5* The Frobeniu norm of the acquired non-diagonal elements can be used to evaluate the similarity (the tool wear state) between the two signals.

## Experimental investigation

To verify the proposed method, an open-source database, and actual milling experiments are employed for the effectiveness verification of the proposed method.

### Investigation in NASA database

As a typical dataset, the NASA Ames and UC Berkeley milling dataset is widely used in the research on the tool condition monitoring of general machining^29^. To investigate the applicability of the proposed method, the NASA dataset is employed in this section. As listed in Table 1, the acoustic emission signals acquired during the experiment (Mstsuura MC-510 V machine center is employed for the experiment) under the spindle speed of 826 rev/min, depth of cut is 0.75 mm, feed speed of 0.25 mm/rev. A 70 mm face mill with 6 inserts is employed for the processing.

**Table 1:** Experiment conditions in NASA database.

Parameter	Spindle speed (rev/min)	Depth of cut (mm)	Feed speed (mm/rev)	Material
Value	826	0.75	0.25	Cast iron

**Figure 4:**
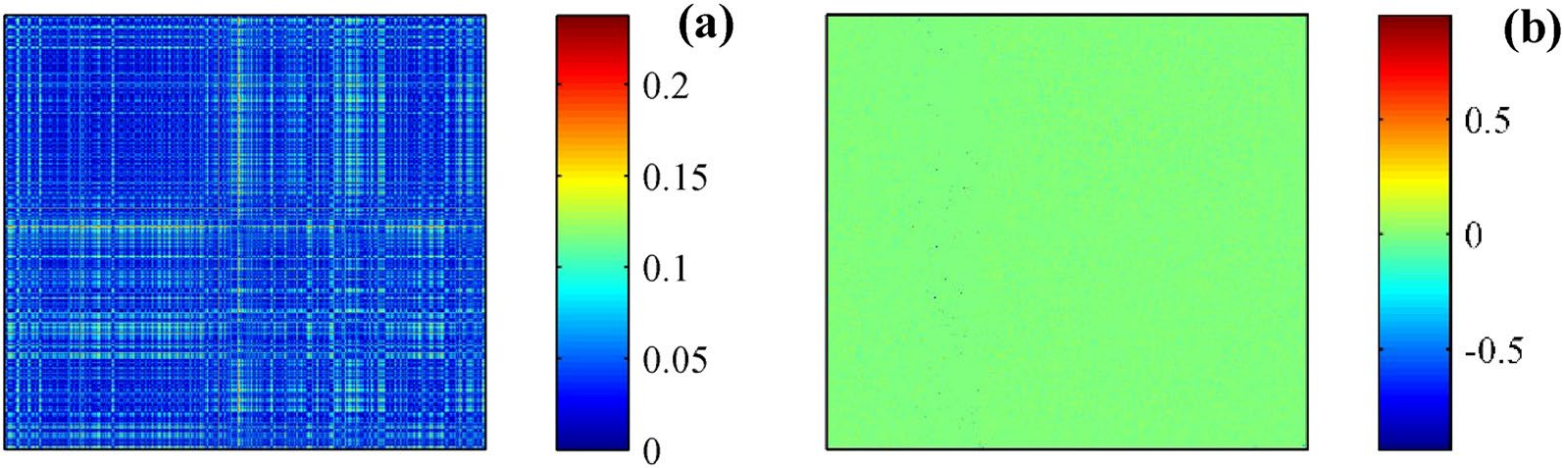
Signal diagonalization of NASA signal. (**a**) Adjacency. (**b**) Eigenvector.

According to the presentation above, appropriate graph structure construction is crucial for tool wear evaluation. The collected time-domain signals contain a significant amount of dynamic information, which can reflect the state of the machining process. Long sampling points can better preserve dynamic information but inevitably affect the timeliness of calculations. Too few sampling points result in the generated graph structure being unable to accurately describe the state information of the machining process. To investigate the performance of tool wear situation in NASA database, setting 800 as the data length of the research, the first sample (data length is 800) is considered as the reference signal (or healthy signal), other samples in the database can be considered as testing signals. Based on the adjacency establish equation before, the calculated reference adjacency is shown in Fig. 4a. Accordingly, the corresponding eigenvalue and eigenvectors (Fig. 4b), columns are the corresponding eigenvectors) are acquired after the diagonalization of the reference adjacency.

**Figure 5:**
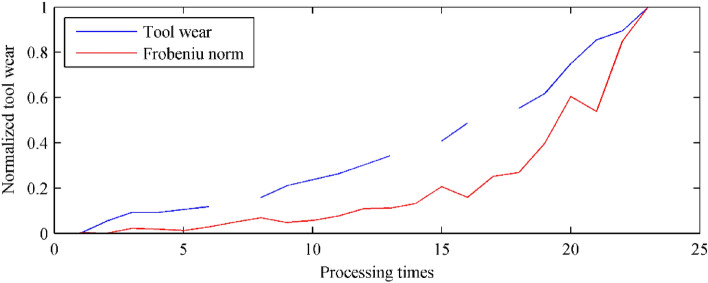
Tool wear evaluation of NASA signal.

Based on the proposed method and the acquired eigenvectors, the fluctuation value of the corresponding signal can be calculated. To evaluate the performance quantitatively, the measured tool wear area and the anomaly are normalized as [0, 1]. The normalization can be represented as:

13 $$ z^{n} = \frac{{{\text{z}} - \min (z)}}{\max (z) - \min (z)}, $$

where *z* indicates the tool wear area or the anomalies sequence, *z*_*n*_ is the normalized sequence, min(⋅), max(⋅) are the minimum and maximum value of the sequence respectively. The evaluated tool wear condition is shown in Table 2. As illustrated in the table, in the whole 23 continuous samples, the corresponding error is ranging from 0 to 0.3280. Accordingly, the mean error between the evaluated tool wear state and the measured tool wear values is calculated as 0.1482. Figure 5 plots the normalized similarity (evaluated tool wear state based on the proposed method, red solid curve in the figure) and the normalized tool wear values (measured tool wear value, blue solid curve in the figure). As shown in Fig. 5, during the milling process, with the deterioration of the tool state, the Frobeniu norm (anomalies) also increased. The two variables had significant simultaneous change trends.

**Table 2:** Tool wear comparison in NASA database.

No.	Measured tool wear	Normalized tool wear	Anomaly	Normalized anomaly	Error
1	0.0000	0.0000	91.5625	0.0033	0.0033
2	0.0400	0.0526	82.5924	0.0000	0.0526
3	0.0700	0.0921	141.2112	0.0219	0.0702
4	0.0700	0.0921	132.3365	0.0186	0.0735
5	0.0800	0.1053	116.5331	0.0127	0.0926
6	0.0900	0.1184	160.5394	0.0291	0.0893
7	–	–	215.4140	0.0495	–
8	0.1200	0.1579	266.2365	0.0685	0.0894
9	0.1600	0.2105	211.9881	0.0483	0.1623
10	0.1800	0.2368	235.6642	0.0571	0.1797
11	0.2000	0.2632	287.4926	0.0764	0.1867
12	0.2300	0.3026	375.9201	0.1094	0.1932
13	0.2600	0.3421	381.3866	0.1115	0.2306
14	–	–	437.7851	0.1325	–
15	0.3100	0.4079	636.0592	0.2065	0.2014
16	0.3700	0.4868	508.3155	0.1588	0.3280
17	–	–	758.6112	0.2522	–
18	0.4200	0.5526	804.4307	0.2693	0.2834
19	0.4700	0.6184	1151.9110	0.3989	0.2195
20	0.5700	0.7500	1703.2531	0.6046	0.1454
21	0.6500	0.8553	1524.5244	0.5379	0.3174
22	0.6800	0.8947	2360.3092	0.8497	0.0450
23	0.7600	1.0000	2763.2240	0.9230	0.0000
				Average	0.1482

### Milling experiment

#### Experiment setup

**Figure 6:**
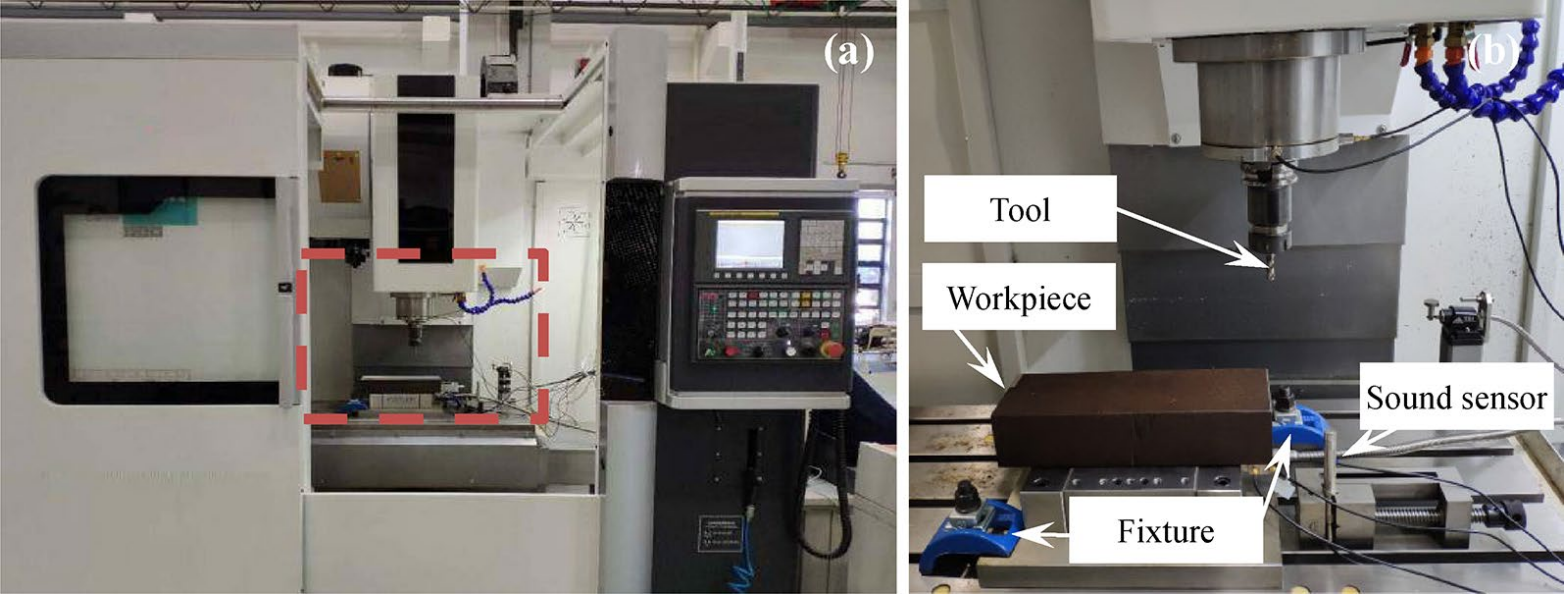
Experiment setup. (**a**) Machine tool. (**b**) Magnification of the marked rectangle area.

**Figure 7:**
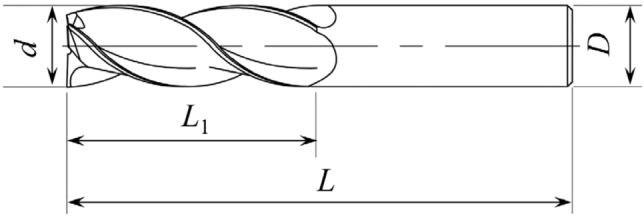
Diagram of the milling cutter.

The experiment setup is shown in Fig. 6. In this experiment, the end milling experiment is conducted on a vertical machining center (Dalian Machine Tool Group DMTG VDL 850A). During the experiment, a kind of uncoated tungsten steel end milling cutter (diameter of 10 mm, detail of the milling cutter can be seen in Fig. 7, Table 3) was employed to cut the workpiece (45 steel, with dimension of 300 × 100 × 80 mm, the chemical properties of the workpiece material is shown in Table 4). Related literatures^30,31^ have shown that there is a certain correlation between the sound pressure signals and the tool wear status during the manufacturing process. To minimize the impact of sensor installation on the machining environment, in this experiment, a non-contact sound pressure sensor is employed to collect dynamic signals during the milling process. During the whole process, the sound pressure sensor (GRAS 46AE, the sensitivity is 50 mV/Pa) is mounted on the table of the machining center near the workpiece (approximately 100 mm away from the workpiece) and used for the acquisition of sound signal. The dynamic signals in this experiment are recorded by a data acquisition instrument (Econ MI-7016 Avant) with a 12 kHz sampling frequency.

**Table 3:** Dimension of the milling cutter.

Parameter	*d*/mm	*D*/mm	*L*_1_/mm	*L*/mm	Helix angle
Value	10	10	30	75	45°

**Table 4:** Chemical properties of the workpiece.

Carbon (C)/%	Silicon (Si)/%	Manganese (Mn)/%	Nickel (Ni)/%	Chromium (Cr)/%	Copper (Cu)/%
0.42–0.50	0.17–0.37	0.50–0.80	< 0.4	< 0.25	< 0.25

**Figure 8:**
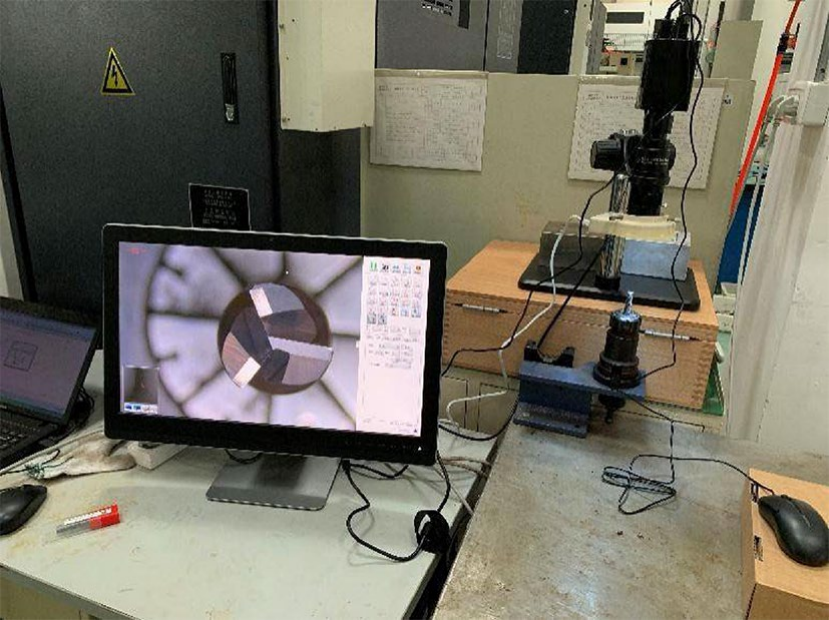
Direct tool wear detection.

**Figure 9:**
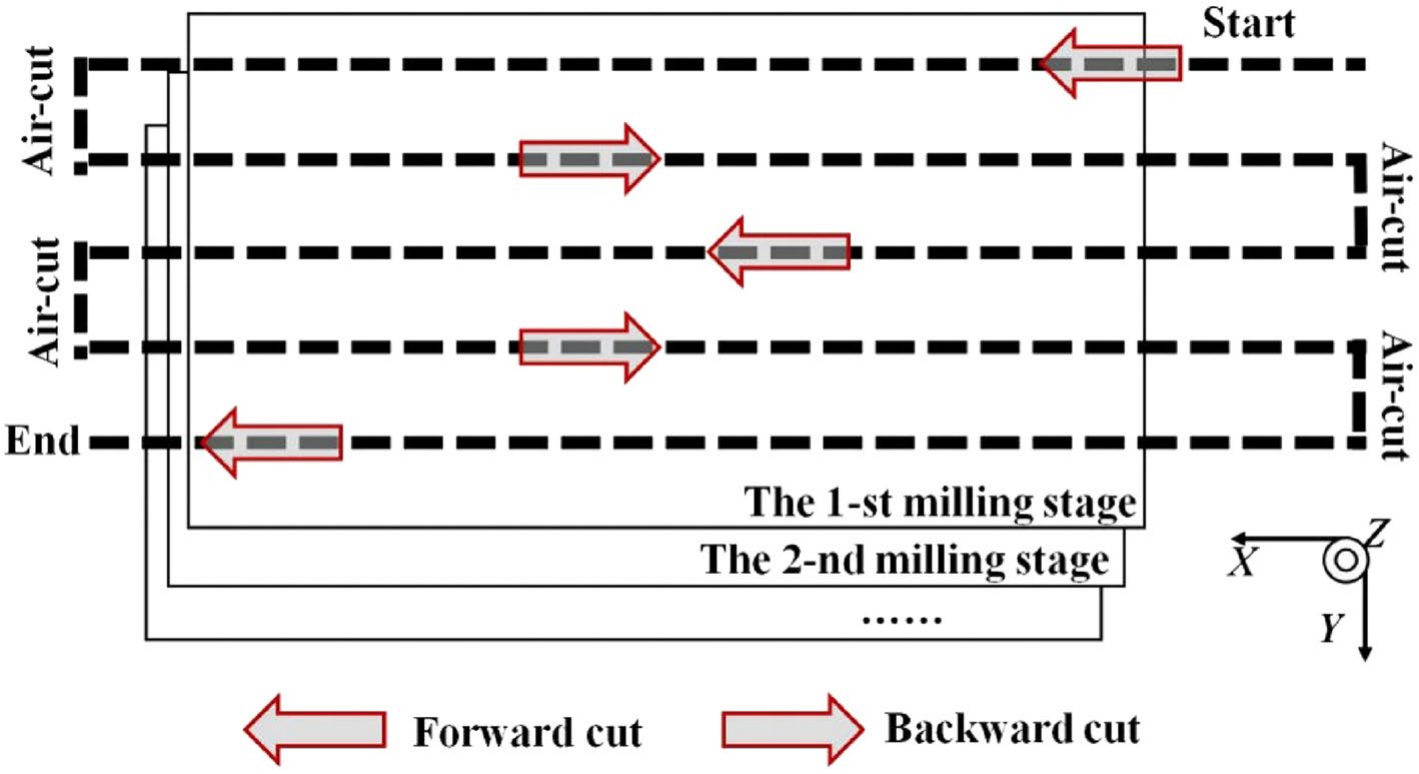
Machining path.

To accurately evaluate the actual tool wear state, a direct measuring instrument (Ksgaopin precision instrument GP-300C, as shown in Fig. 8) is employed for the estimation. Obviously, the three teeth are independent, the evaluation process of each tooth should perform separately. In the experiment process, the workpiece is manufactured layer upon layer. There are three forward and two backward cuts in each layer, as described in Fig. 9. After finishing one layer of the workpiece, the tool holder is taken off to evaluate the tool wear state. Generally, according to ISO3685-1977, the tool wear state is presented as the tool flank wear VB. However, as mentioned in related literature, the one-dimensional evaluation parameter cannot fully reflect the tool wear state. In this research, the mean flank wear area of the three flanks is indicated for the tool wear state evaluation.

#### Tool wear evaluation

In order to validate the reliability in actual milling experiments, two milling tests were conducted with the experimental setup. The experimental conditions are shown in Table 5.

**Table 5:** Experiment conditions in milling tests.

	Spindle speed (rev/min)	Depth of cut (mm)	Feed speed (mm/min)	Material
Case I	2500	0.6	400	45# steel
Case II	2500	0.4	400	45# steel

##### Case I

**Figure 10:**
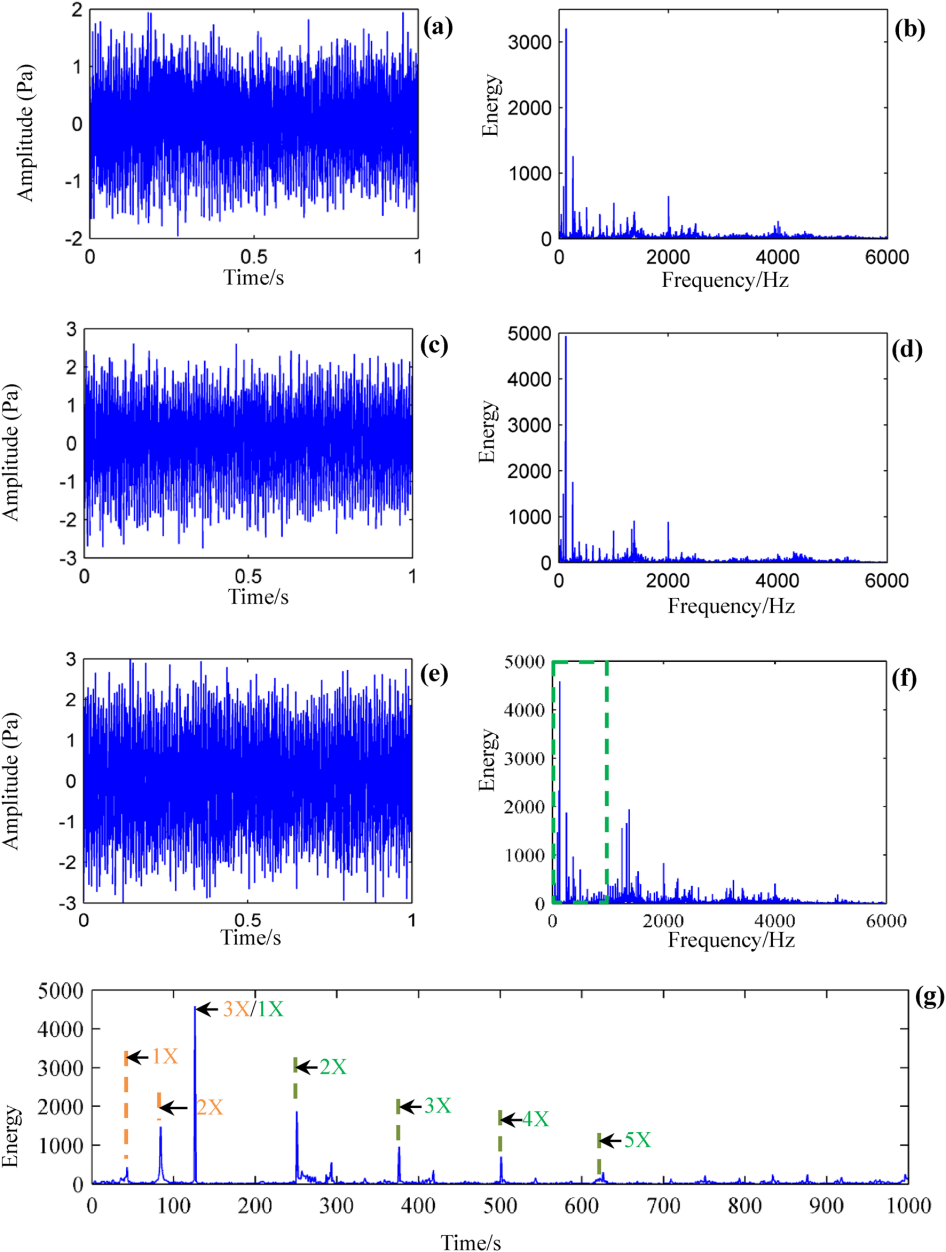
Signal samples in the milling process. (**a**) Time-domain signal of the 1st layer. (**b**) Frequency spectrum of the 1st layer. (**c**) Time-domain signal of the 3rd layer. (**d**) Frequency spectrum of the 3rd layer. (**e**) Time-domain signal of the 5th layer. (**f**) Frequency spectrum of the 5th layer. (**g**) Local magnification of (**f**).

Figure 10 plots the waveforms of the acquired acoustic signals via the sound sensor, as well as the frequency domain. The sampled signals in the 1st layer, 3rd layer, and 5th layer are described in Fig. 10a,c,e, respectively. The corresponding frequency spectrums are listed in Fig. 10b,d,f, respectively. As can be seen in the figure, there is almost no obvious variation law or characteristics between the two samples either in time-domain waveforms or frequency-domain. The local magnification of the frequency spectrum (green dashed rectangle in Fig. 10f) of the 5th layer is shown in Fig. 10g. The spindle speed during the milling process is 2400 rev/min. Therefore, the spindle rotating frequency (40 Hz) and its harmonics can be observed (orange dashed lines). Caused by the distributed three teeth, the cutting frequency (120 Hz) and its harmonics can also be monitored (green dashed lines).

**Figure 11:**
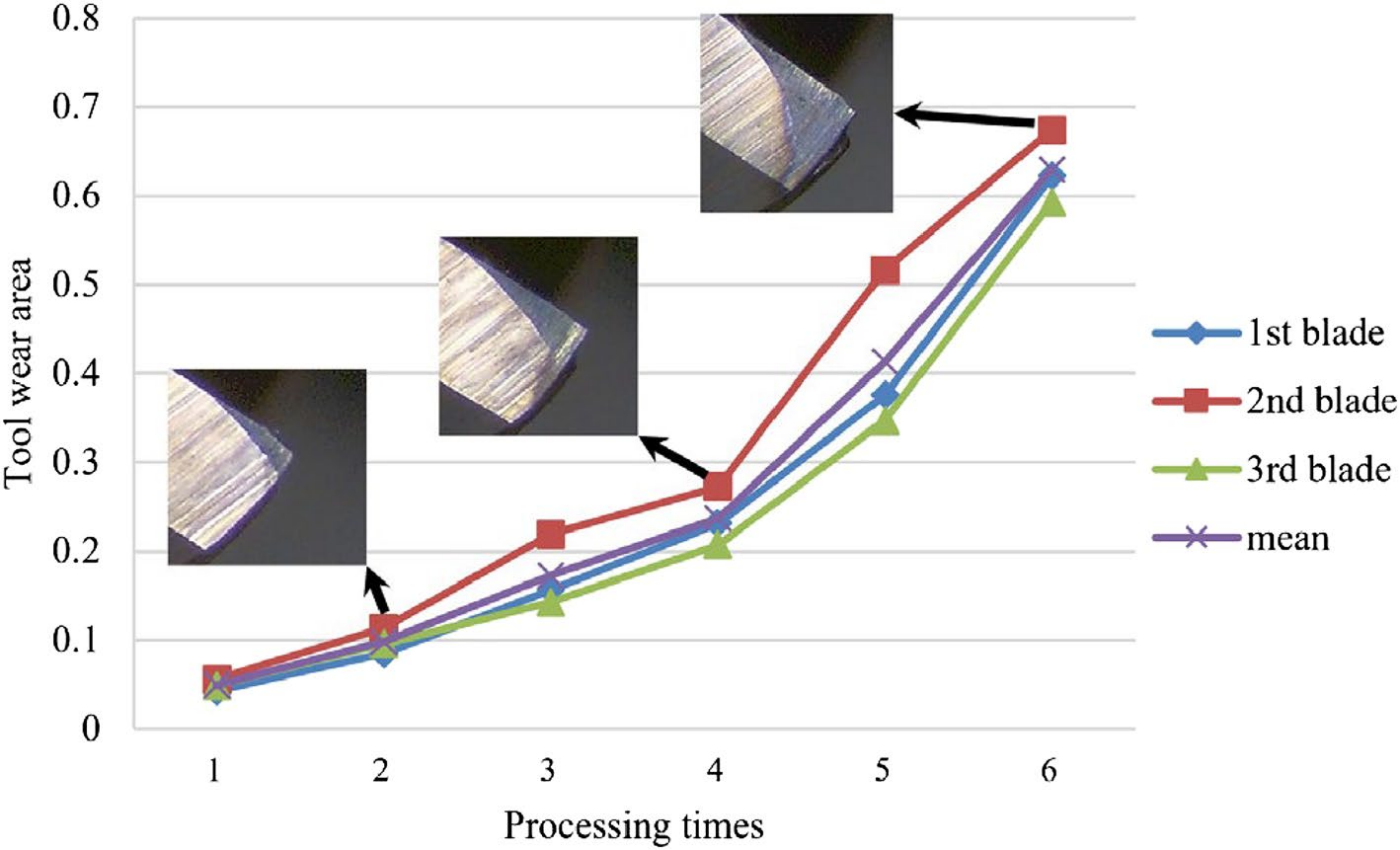
Tool wear variation in milling Case I.

With the mentioned direct measuring instrument, the actual tool wear state during the manufacturing process can be monitored. Generally, the evolution of wear in tool experiments is a continuous process. The variation process of the wear area and its average value of three cutting edges is shown in Fig. 11, as well as the tool wear images. According to Fig. 11, caused by direct contact with the cutting material, generally, a triangular wear band will occur at the cutter edge tip. Besides, the maximum flank wear will also tend to appear around the tooltip. The specific information during the milling tool wear evolution process is shown in Table 6. As can be seen in Fig. 11 and Table 6, with the increase in cutting time, the tool wear area grew to mm^2^ (mean tool wear area).

**Table 6:** Tool wear area in milling Case I.

Layer no.	Tool wear area/mm^2^			
	1st blade	2nd blade	3rd blade	Mean
1	0.0117	0.0171	0.0059	0.0116
2	0.0288	0.0406	0.0139	0.0278
3	0.0588	0.0727	0.0390	0.0568
4	0.1166	0.1403	0.0900	0.1156
5	0.2212	0.2090	0.1398	0.1900
6	0.2804	0.3301	0.2572	0.2892

**Figure 12:**
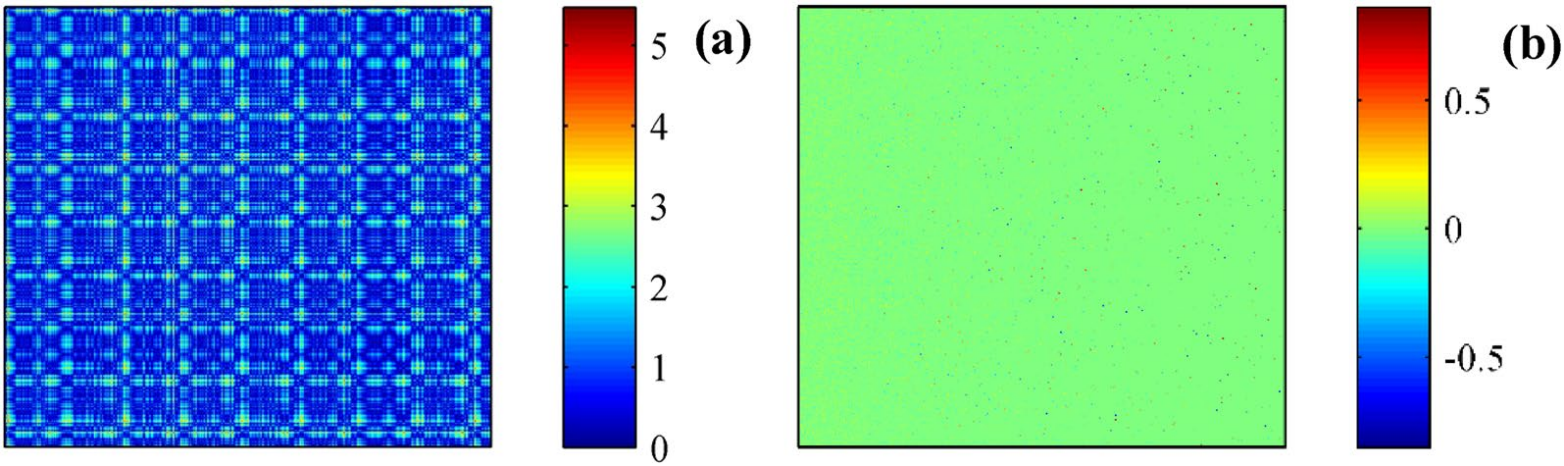
Signal diagonalization of the first sample in Case I. (**a**) Adjacency. (**b**) Eigenvector.

By considering the first sample (still setting the data length as 800) is considered as the reference signal (or healthy signal), the other samples can be considered as testing signals. Based on the adjacency establish equation before, the calculated reference adjacency is shown in Fig. 12a. Accordingly, the corresponding eigenvalue and eigenvectors (Fig. 12b, columns are the corresponding eigenvectors) are acquired after the diagonalization of the reference adjacency.

**Figure 13:**
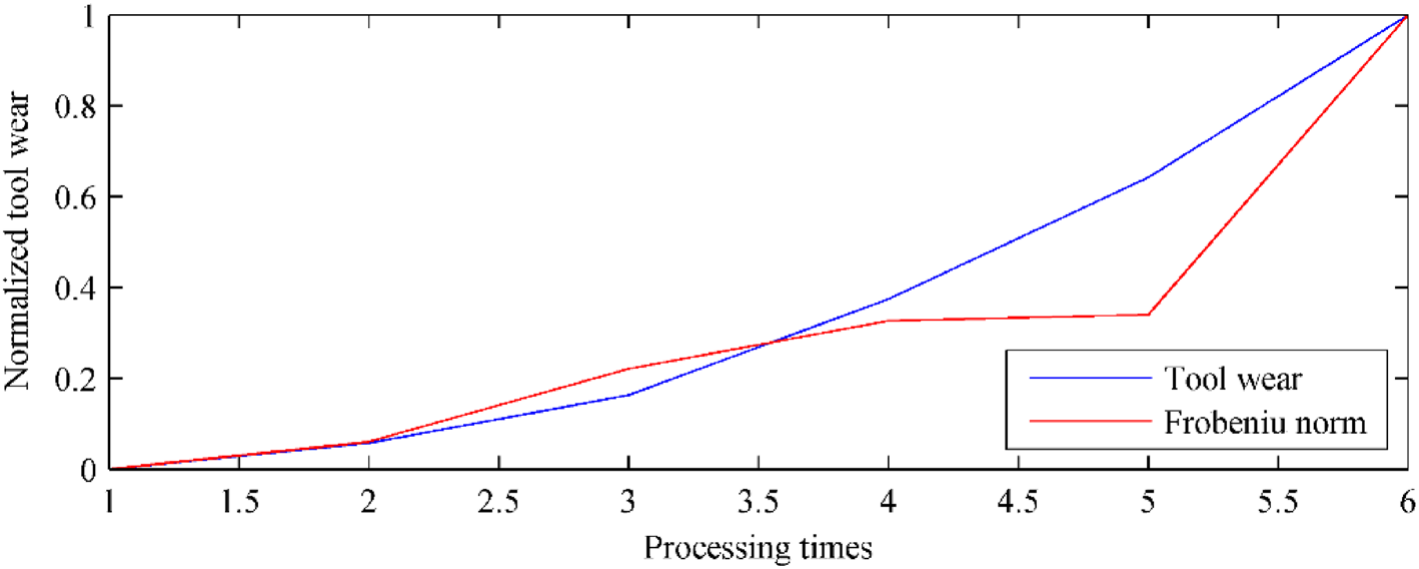
Tool wear evaluation in Case I.

Based on the proposed method and the acquired eigenvectors, the fluctuation can be calculated for the similarity evaluation according to Eq. (12). The measured tool wear states, the anomalies, and their errors are summarized in Table 7. As illustrated in the table, in the whole 6 continuous samples, the corresponding error is ranging from 0 to 0.2350. Accordingly, the mean error between the evaluated tool wear state and the measured tool wear values is calculated as 0.0881. Figure 13 plots the normalized similarity (evaluated tool wear state based on the proposed method, blue solid curve in the figure) and the normalized tool wear values (measured tool wear value, red solid curve in the figure). The slimier simultaneous trend indicates the potential mapping relationship between the Frobeniu norm (anomalies) and the tool wear.

**Table 7:** Tool wear comparison in Case I.

No.	Measured tool wear	Normalized tool wear	Anomaly	Normalized anomaly	Error
1	0.0116	0.0000	53,183	0.0000	0.0000
2	0.0278	0.0583	64,770	0.0604	0.0050
3	0.0568	0.1630	95,596	0.2210	0.1249
4	0.1156	0.3748	115,877	0.3267	0.1638
5	0.1900	0.6426	118,475	0.3402	0.2350
6	0.2892	1.0000	245,106	1.0000	0.0000
				Average	0.0881

##### Case II

**Figure 14:**
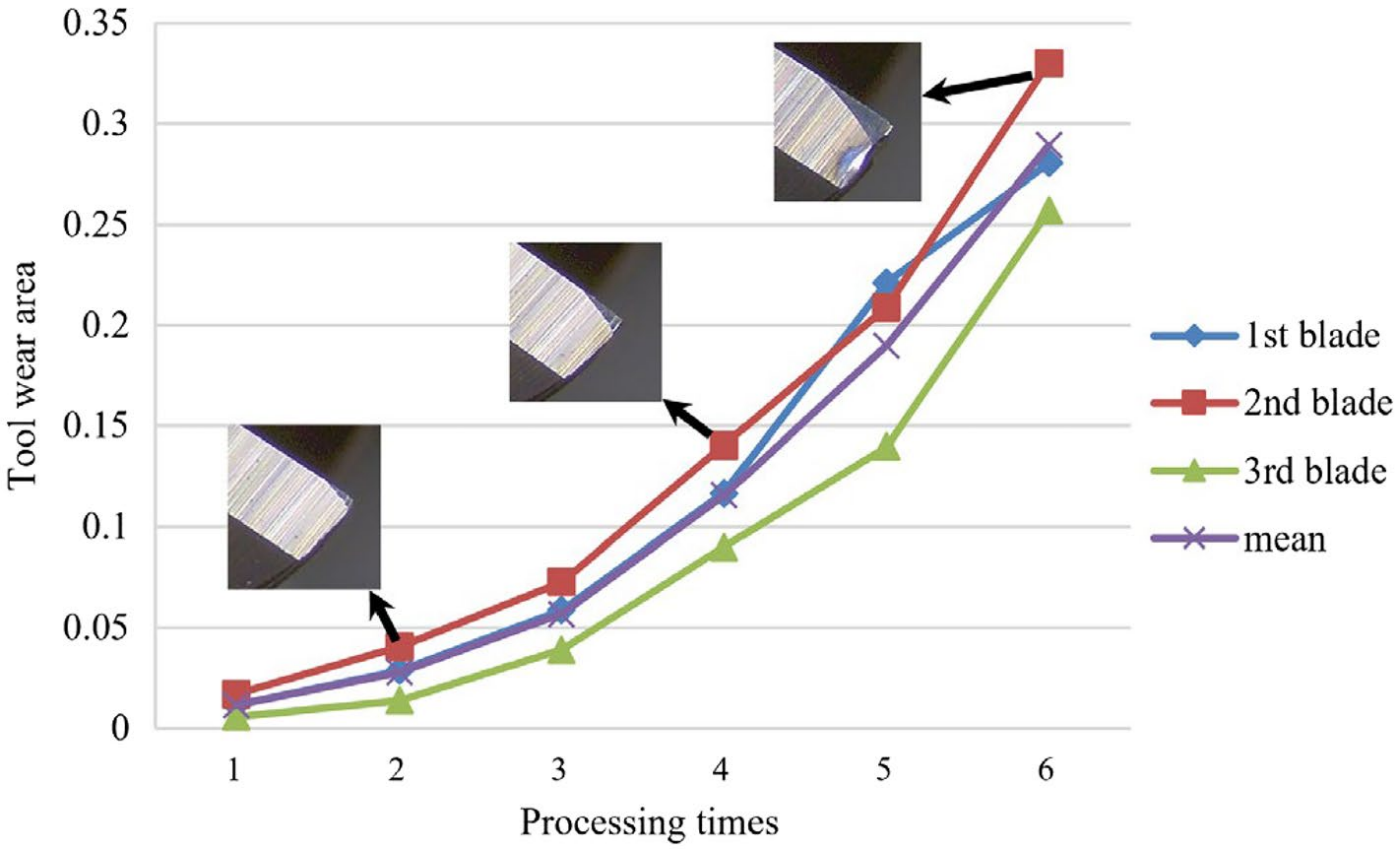
Tool wear variation in milling Case II.

The variation process of the wear area and its average value of three cutting edges is shown in Fig. 14, as well as the tool wear images. The specific information during the milling tool wear evolution process is shown in Table 8. As can be seen in Fig. 14 and Table 8, with the increase in cutting time, the tool wear area grew to mm^2^ (mean tool wear area).

**Table 8:** Tool wear area in milling Case II.

Layer no.	Tool wear area/mm^2^			
	1st blade	2nd blade	3rd blade	Mean
1	0.0429	0.0556	0.0486	0.0490
2	0.0845	0.1136	0.0955	0.0979
3	0.1567	0.2189	0.1426	0.1727
4	0.2311	0.2729	0.2069	0.2370
5	0.3754	0.5164	0.3470	0.4129
6	0.6223	0.6741	0.5932	0.6299

**Figure 15:**
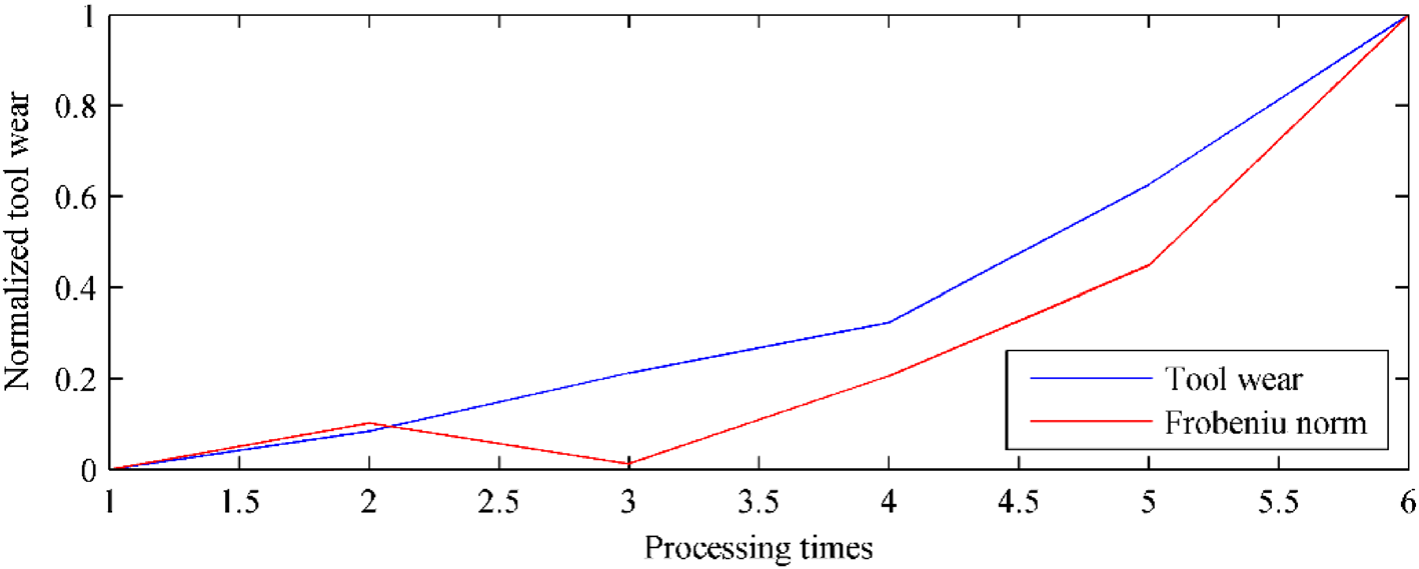
Tool wear evaluation in Case II.

With the same investigation method mentioned before, the measured tool wear states, the anomalies, and their errors can be calculated, as listed in Table 9. As illustrated in the table, in the whole 6 continuous samples, the corresponding error ranges from 0 to 0.1266 (the measured tool wear values is calculated as 0.0484). Figure 15 plots the normalized similarity (evaluated tool wear state based on the proposed method, blue solid curve in the figure) and the normalized tool wear values (measured tool wear value, red solid curve in the figure).

**Table 9:** Tool wear comparison in Case II.

No.	Measured tool wear	Normalized tool wear	Anomaly	Normalized anomaly	Error
1	0.0490	0.0000	27,976	0.0000	0.0000
2	0.0979	0.0841	44,789	0.1025	0.0372
3	0.1727	0.2130	30,021	0.0125	0.0836
4	0.2370	0.3236	61,737	0.2059	0.0430
5	0.4129	0.6265	101,547	0.4486	0.1266
6	0.6299	1.0000	191,960	1.0000	0.0000
				Average	0.0484

#### Tool wear evaluation in different data length

As presented above, data length during the data processing process is crucial for the tool wear evaluation. To investigate the influence of data length in the evaluation process, repeat measuring experiments are conducted. The results in different data lengths are shown in Table 10. As can be conducted in the table, when the data length varies from 100 to 1000, the evaluation error distribution in the two experiments has no obvious change regular pattern. According to the results, the mean error for the two experiments reaches its minimum value when the data length is 800. Therefore, in this research, the data length is set as 800.

**Table 10:** Tool wear evaluation error in different length.

No.	Length	Error			
		NASA dataset	Milling data (Case I)	Milling data (Case II)	Mean
1	100	0.1509	0.2019	0.1956	0.1828
2	200	0.1464	0.1569	0.2350	0.1794
3	300	0.1442	0.1319	0.0915	0.1225
4	400	0.1602	0.1810	0.2155	0.1856
5	500	0.1500	0.1299	0.1015	0.1271
6	600	0.1529	0.2450	0.1233	0.1737
7	700	0.1511	0.1838	0.1415	0.1588
8	800	0.1482	0.0881	0.0484	0.0949
9	900	0.1420	0.0931	0.0996	0.1116
10	1000	0.1499	0.1241	0.1079	0.1273

## Conclusion

Aiming to the quantitative evaluation of tool wear state, in this research, authors proposed a two-stage method to estimate the tool running condition directly from the time series. In the prediction stage, with the engagement of dynamic mode decomposition, the real-time response of the end milling process can be predicted. In the estimation process, by constructing the graph structure of the time series and calculating the difference between the predicted signal and the real-time signal, the tool wear state during the end milling process can be successfully evaluated. To further confirm the effectiveness of the proposed method, investigations in an open source are presented and achieve a preferable effect. The results were also confirmed by an actual milling experiment from our laboratory. Accordingly, the combination of dynamic mode decomposition and anomalies evaluation method presents a wide range of possibilities for the further development of condition monitoring and fault detection techniques via time series. Besides, in this research, it is assumed that there is a linear relationship between the anomalies and the real tool wear. The nonlinear factors caused by environmental factors such as process parameters and tools have not been considered. Further mechanistic studies and the development of the proposed method are still ongoing in our team.

## Data Availability

The data that support the findings of this study are included and will be available from the corresponding author upon reasonable request.

## References

[1] Pimenov DY, Bustillo A, Wojciechowski S, Sharma VS, Gupta MK, Kuntoğlu M (2023). Artificial intelligence systems for tool condition monitoring in machining: Analysis and critical review. J. Intell. Manuf..

[2] Lin WJ, Chen JW, Jhuang JP, Tsai MS, Hung CL, Li KM, Young HT (2021). Integrating object detection and image segmentation for detecting the tool wear area on stitched image. Sci. Rep..

[3] Wojciechowski S, Twardowski P (2012). Tool life and process dynamics in high speed ball end milling of hardened steel. Procedia CIRP.

[4] Wojciechowski S, Krajewska-Śpiewak J, Maruda RW, Krolczyk GM, Nieslony P, Wieczorowski M, Gawlik J (2023). Study on ploughing phenomena in tool flank face—Workpiece interface including tool wear effect during ball-end milling. Tribol. Int..

[5] Zhou Y, Sun B, Sun W (2020). A tool condition monitoring method based on two-layer angle kernel extreme learning machine and binary differential evolution for milling. Measurement.

[6] Lei Z, Zhu Q, Zhou Y, Sun B, Sun W, Pan X (2021). A GAPSO-enhanced extreme learning machine method for tool wear estimation in milling processes based on vibration signals. Int. J. Precis. Eng. Manuf. Green Technol..

[7] Zi X, Gao S, Xie Y (2024). An online monitoring method of milling cutter wear condition driven by digital twin. Sci. Rep..

[8] Yang C, Zhou J, Li E, Wang M, Ting J (2022). Local-feature and global-dependency based tool wear prediction using deep learning. Sci. Rep..

[9] Deng W, Zhang S, Zhao S, Yang X (2019). A novel fault diagnosis method based on improved empirical wavelet transform and maximum correlated kurtosis deconvolution for rolling element bearing. J. Mech. Eng..

[10] McDonald GL, Zhao Q (2017). Multipoint optimal minimum entropy deconvolution and convolution fix: Application to vibration fault detection. Mech. Syst. Signal Process..

[11] Liu Y, Xiang H, Jiang Z, Xiang J (2023). Iterative synchrosqueezing-based general linear chirplet transform for time-frequency feature extraction. IEEE Trans. Instrum. Meas..

[12] Ruan D, Han J, Yan J, Gühmann C (2023). Light convolutional neural network by neural architecture search and model pruning for bearing fault diagnosis and remaining useful life prediction. Sci. Rep..

[13] Wu T, Yao Y, Li Z, Chen B, Wu Y, Sun W (2024). Remaining useful life prediction of circuit breaker operating mechanisms based on wavelet-enhanced dual-tree residual networks. J. Power Electron..

[14] Zhi G, He D, Sun W, Zhou Y, Pan X, Gao C (2021). An edge-labeling graph neural network method for tool wear condition monitoring using wear image with small samples. Meas. Sci. Technol..

[15] Mishra D, Awasthi U, Pattipati KR, Bollas GM (2023). Tool wear classification in precision machining using distance metrics and unsupervised machine learning. J. Intell. Manuf..

[16] Li X, Zhou S, Wang F, Fu L (2024). An improved sparrow search algorithm and CNN-BiLSTM neural network for predicting sea level height. Sci. Rep..

[17] Dutta A, McKay M, Kopsaftopoulos F, Gandhi F (2021). Statistical residual-based time series methods for multicopter fault detection and identification. Aerosp. Sci. Technol..

[18] Wang T, Liu Z, Lu G, Liu J (2020). Temporal-spatio graph based spectrum analysis for bearing fault detection and diagnosis. IEEE Trans. Ind. Electron..

[19] Yang C, Liu J, Zhou K, Li X (2024). Dynamic spatial-temporal graph-driven machine remaining useful life prediction method using graph data augmentation. J. Intell. Manuf..

[20] Sun W, Zhou Y, Xiang J, Chen B, Feng W (2021). Hankel matrix-based condition monitoring of rolling element bearings: An enhanced framework for time-series analysis. IEEE Trans. Instrum. Meas..

[21] Feng S, Wang Z, Jiang W, Chen B, Yuan Z, Sun W (2023). A time-series driven mechanical system state description method and its application in condition monitoring. IEEE Sens. J..

[22] Kutz JN, Brunton SL, Brunton BW, Proctor JL (2016). Dynamic Mode Decomposition: Data-Driven Modeling of Complex Systems.

[23] Dai L, Cao W, Yi S, Wang L (2023). Damage identification of concrete structure based on WPT-SVD and GA-BPNN. J. Zhejiang Univ. (Eng. Sci.).

[24] Yang J, Shen L, Zheng Z, Li T, Yang Y (2023). Transmission tower looseness detection based on dynamic mode decomposition. J. Vib. Shock.

[25] Zhang F, Liu J, Li Y, Liu Y, Ge MF, Jiang X (2023). A health condition assessment and prediction method of Francis turbine units using heterogeneous signal fusion and graph-driven health benchmark model. Eng. Appl. Artif. Intell..

[26] Liu J, Zhou K, Yang C, Lu G (2021). Imbalanced fault diagnosis of rotating machinery using autoencoder-based SuperGraph feature learning. Front. Mech. Eng..

[27] Wen X, Lu G, Liu J, Yan P (2020). Graph modeling of singular values for early fault detection and diagnosis of rolling element bearings. Mech. Syst. Signal Process..

[28] Sun W, Zhou Y, Cao X, Chen B, Feng W, Chen L (2020). A two-stage method for bearing fault detection using graph similarity evaluation. Measurement.

[29] Goebel, A. A. A. K. *Best Lab, UC Berkeley Milling Data Set* (NASA Ames Research Center).

[30] Liu M, Tseng Y, Tran M (2019). Tool wear monitoring and prediction based on sound signal. Int. J. Adv. Manuf. Technol..

[31] Li Z, Liu X, Incecik A, Gupta MK, Królczyk GM, Gardoni P (2022). A novel ensemble deep learning model for cutting tool wear monitoring using audio sensors. J. Manuf. Process..

